# Graphene-Based Polarization-Independent Mid-Infrared Electro-Absorption Modulator Integrated in a Chalcogenide Glass Waveguide

**DOI:** 10.1186/s11671-021-03538-7

**Published:** 2021-05-08

**Authors:** Yong Zhou, Rongguo Lu, Guangbiao Wang, Jiangbo Lyu, Meng Tan, Liming Shen, Rui Lin, Zhonghua Yang, Yong Liu

**Affiliations:** 1 School of Optoelectronic Science and Engineering, University of Electronic Science and Technology of China, Chengdu, 610054 China; 2 Chongqing United Microelectronics Center, Chongqing, 401332 China

**Keywords:** Chalcogenide glass, Graphene, Mid-infrared, Optical-modulator, Polarization-insensitive

## Abstract

A polarization-insensitive graphene-based mid-infrared optical modulator is presented that comprised SiO_2_/ Ge_23_Sb_7_S_70_, in which two graphene layers are embedded with a semiellipse layout to support transverse magnetic (TM) and transverse electric (TE) polarizing modes with identical absorption. The key performance index for the polarization independent modulator is polarization-sensitivity loss (PSL). The waveguide of our device just supports basic TE and TM modes, and the PSL between two modes is of < 0.24 dB. The model can offer extinction ratio (ER) more than 16 dB and insertion loss less than 1 dB. The operation spectrum ranges from 2 to 2.4 μm with optical bandwidth of 400 nm. The 3 dB modulation bandwidth is as high as 136 GHz based on theoretical calculation.

## Introduction

Near-infrared wavelength optical fiber communication networks are becoming the core of the whole telecommunication networks. However, the mid-infrared is also an important waveband for the application of electro-optic device in military and civil fields, such as infrared countermeasure, chemical sensing, infrared guidance, environmental monitoring, space communication, etc. In addition, mid-infrared integrated electro-optic devices, such as photodetectors and modulators, also are developed to expand the 1.55 μm communication window.

In recent years, 2D functional electro-optic materials, such as graphene [1–4], chalcogenide [5], and black phosphorus [6], have been discovered, which accelerated the development of integrated electro-optic and broke the traditional performance limitation. Among these materials, graphene is considered as an ideal material for realizing optical modulators due to some attractive advantages [7], such as constant absorption over a wide spectrum [8], ultra-high carrier mobility at room temperature [9], electrically controllable conductivity and compatibility with CMOS processing. Consequently, graphene-based optical modulator has become a hot research topic. However, by far, the operation waveband of most reported graphene-based optical modulators is around 1.31 μm or 1.55 μm [10–13]. The modulation principle of near infrared and mid-infrared is the same, but the operation wavelength of modulator mainly depends on the waveguide transparency windows. The key point for the realization of graphene-based mid-infrared modulators is the integration of graphene and various mid-infrared waveguide materials. In 2017, Lin et al. [14] reported a mid-infrared electro-absorption optical modulator based on Ge_23_Sb_7_S_70_-on-graphene structure, which opened the field of graphene-based mid-infrared modulator.

Graphene as electro-optic material, we also need to consider one of the most important characteristics of anisotropic dielectric [15], which has been experimentally proved in this article [16]. The permittivity in plane is tunable, however, the permittivity in vertical is a constant of 2.5. So, the graphene can only strongly interact with the in-plane electric field [10], that is the reason why reported graphene-based modulators before have a strong polarization dependence, in which modulators can only modulate in-plane electric filed mode [10–13]. Generally, the polarization state of light in waveguide or fiber is random. To realize the wide commercial application of graphene-based modulator, the problem of polarization dependent needs to be solved.

In this work, we present a new structure of graphene-based mid-infrared polarization-independent electro-optic modulator, which has the advantages of large modulation bandwidth and wide spectrum of polarization insensitivity. We used the SOI structure and a Ge_23_Sb_7_S_70_ glass strip which is embedded in SiO_2_ cladding as waveguide core. In the Ge_23_Sb_7_S_70_ waveguide, two graphene layers are U (semiellipse)-type distribution and are insulated by Ge_23_Sb_7_S_70_ glass. Since the graphene layer is U-type distribution, both TE and TM modes can strongly interact with graphene. By proper choosing structure parameters, we can well overcome the polarization dependence. Using the finite element method (FEM), we analyzed the effective mode index (EMI) and absorption coefficient (*α*) of the *U*-structure device. The result shows that the real parts of EMI for TE (*N*_te_) and TM (*N*_tm_) modes have the same fluctuations (with constant difference) in different chemical potential (*μ*_c_), and the imaginary parts of both TE and TM modes have almost identical fluctuations and wavelength independent in a wide spectrum. By proper choosing of switching points for “ON” and “OFF” states, for both TE and TM modes, the modulation depth is more than 16 dB, the operation wavelength spectrum is 2–2.4 μm, the PSL is less than 0.24 dB, and the theoretical 3 dB modulation bandwidth is as high as 136 GHz.

## Methods

**Fig. 1:**
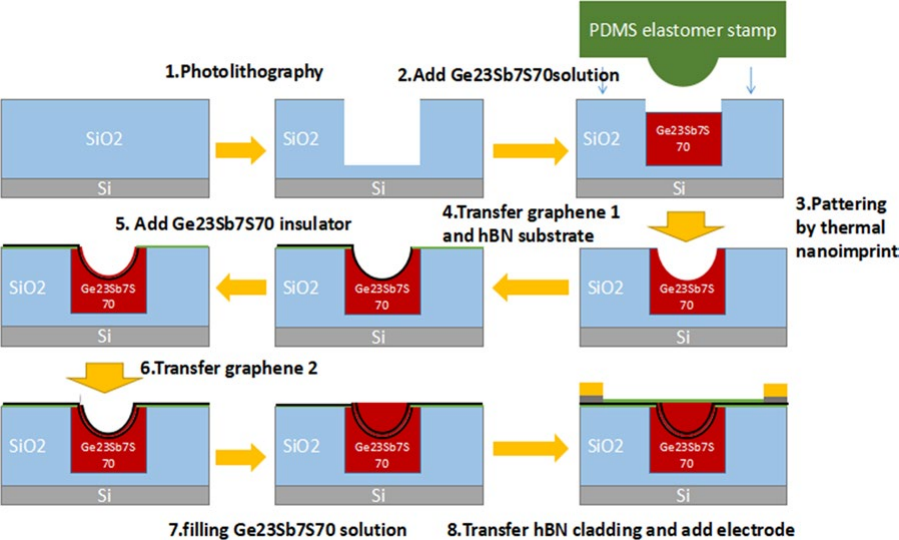
Schematic process flow of the graphene-based modulator integrated in Ge_23_Sb_7_S_70_

**Fig. 2:**
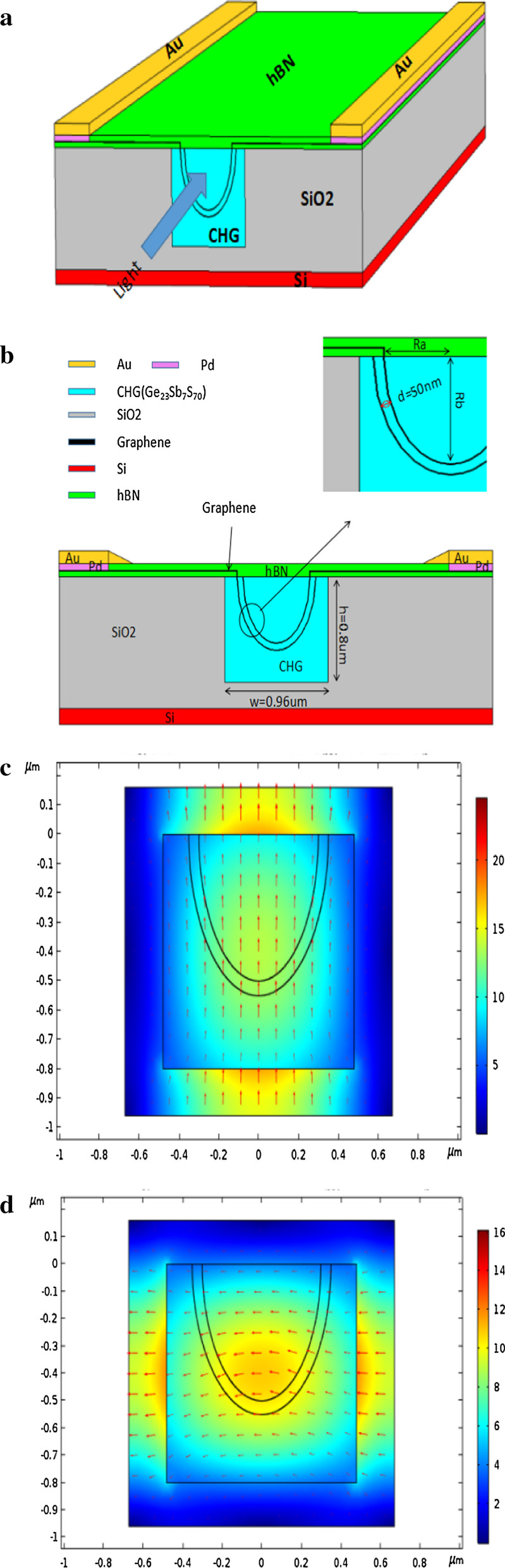
Illustration of the polarization-independent electro-absorption optical modulator. **a** 3D Schematic diagram of the modulator; **b** 2D cross section of the U-structure Ge_23_Sb_7_S_70_-graphene waveguide, distance between two graphene layer *d* = 50 nm, waveguide width *w* = 0.96 μm, height *h* = 0.8 μm. The electric field distribution for TE mode (**c**) and TM mode (**d**), arrows indicate the direction of polarization

The transparency window of Ge_23_Sb_7_S_70_ glass ranges from 2 to 10 μm [17] that is a great material for mid-infrared photonics. Previous study led by Lin et al. [14] has proved its feasibility to realize Ge_23_Sb_7_S_70_-graphene mid-infrared modulator. In this work, we also take Ge_23_Sb_7_S_70_ glass as waveguide material. The geometrical structure of our proposed modulator is depicted in Fig. 1, which was fabricated using a thermal nanoimprint process. Details of the process steps are schematically illustrated in Fig. 1. You can also reference paper [18] to get details for the preparation of PDMS composite stamps and Ge_23_Sb_7_S_70_ glass solution. Details for geometrical size and materials are presented in Fig. 2b.

A SiO_2_ layer with thickness h = 0.8 μm was grown on Si substrate, and then a groove with width w = 0.96 μm and height h = 0.8 μm was made in SiO_2_ layer by using photolithography method. After filling Ge_23_Sb_7_S_70_ solution and pattering by thermal nanoimprint, a U-type Ge_23_Sb_7_S_70_ groove was made. A 10-nm-thickness hexagonal boron nitride (hBN) layer was paved at flat area. Then, first graphene layer, 50-nm-thickness (spin-coating) Ge_23_Sb_7_S_70_ insulator and second graphene layer were paved to the U-type Ge_23_Sb_7_S_70_ groove in order. Finally, we filled the U-type Ge_23_Sb_7_S_70_ groove with Ge_23_Sb_7_S_70_ solution and transferred hBN cladding and added electrode. The electrode structure is Au–Pd-graphene since the contact resistance between graphene and Pd is less than 100(Ω/μm) [19]. Graphene sheet width between electrode and waveguide is 0.8 μm. Figure 2c, d presents the electric field distribution for both TE (in-plane) and TM (vertical-plane) modes.

When voltage is applied onto the graphene, graphene’s chemical potential *μ*_c_ is dynamically tuned. In our model, graphene is treated as an anisotropic material. The perpendicular permittivity *ε*_*⊥*_ of the graphene does not vary with the *μ*_c_ and always stays as a constant of 2.5, whereas the in-plane permittivity of the graphene *ε*_‖_ can be tuned as [12].

1 $$\varepsilon_{\parallel } \left( \omega \right) = 1 + \frac{i\delta }{{\omega \varepsilon_{0} h_{g} }}$$

The *δ* stands for the conductivity of graphene and relates to chemical potential *μ*_c_, which can be deduced from Kubo formula [20]. The *ω* represents the radian frequency, and *h*_*g*_ = 0.7 nm is the effective thickness of graphene.

**Fig. 3:**
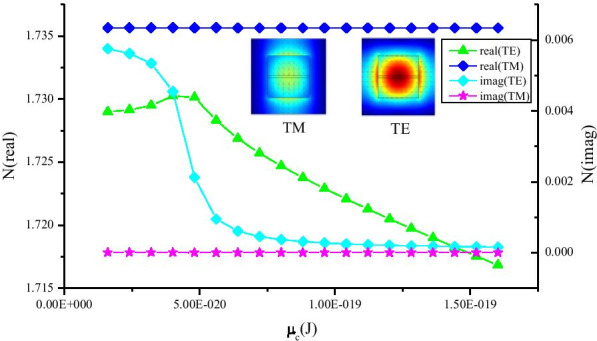
Graphene was straightly paved in Ge_23_Sb_7_S_70_ strip waveguide. The real and imaginary parts of EMI for both TE and TM modes at the wavelength of 2.2 μm

We made a Ge_23_Sb_7_S_70_ strip waveguide, in which two flat graphene layers were embedded (Fig. 3 insert). Figure 3 plots the real and imaginary part of EMI for both TE and TM mode at the wavelength of 2.2 μm. The EMI of TE mode changed obviously for both real and imaginary parts. On contrary, no significant fluctuations occurred to the EMI of TM mode for both real and imaginary parts. The main reason is that TM mode polarization is perpendicular to the graphene plane and *ε*_‖_ is nontunable in chemical potential. In this work, we bend the graphene layer as U-type layout to impose equal influence on both TE and TM modes.

## Results and Discussion

**Fig. 4:**
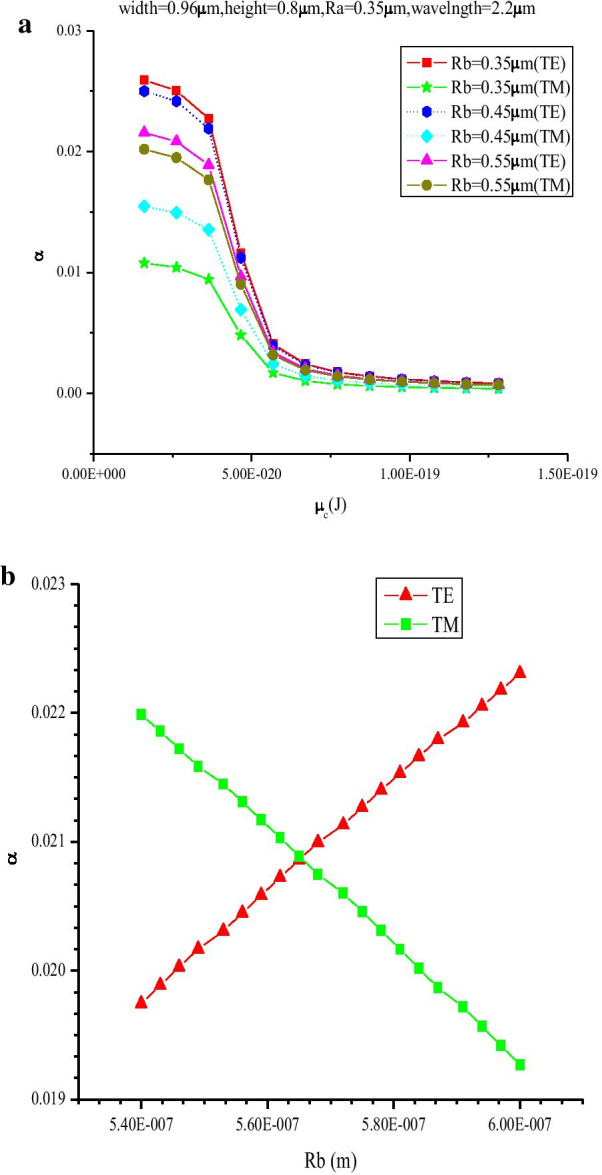
**a** Absorption coefficient of TE and TM modes as a function of *μ*_c_ at different Rb, (wavelength = 2.2 μm, Ra = 0.35 μm); **b** the absorption coefficient of TE and TM modes as a function of Rb (Ra = 0.35 μm, wavelength = 2.2 μm, *μ*_c_ = 0.1 eV)

Although the polarization-independent electro-optic modulator based on graphene has been reported [15–21], the polarization independence of these devices is closely related to wavelength [22]. Therefore, in our model, the U-structure is used, in which we find that the sensitivity of the waveguide polarization is weak correlation with wavelength. The imaginary part of the EMI is known as electro-absorption. As shown in Fig. 3, the imaginary part of the EMI reaches peak at low chemical potential around *μ*_c_ = 0.1 eV. Thus, the *μ*_c_ = 0.1 eV point can be chosen as “OFF” state point. At the same time, the discrepancy of imaginary part of the EMI between TE and TM modes is highest at “OFF” state point. To get lower discrepancy of absorption, we just need to minimize the discrepancy of absorption at “OFF” state point. At wavelength = 2.2 μm and Ra = 0.35 μm (size of minor radius of the ellipse that is the horizontal axis), by sweeping the *μ*_c_ from 0.1 to 0.8 eV, under different Rb (size of major radius of the ellipse that is the vertical axis), the influence of varied *μ*_c_ on EMI for both TE and TM modes is analyzed, as shown in Fig. 4a. It is obvious that the discrepancy values between the TE and TM modes decrease rapidly as Rb is tuned from 0.35 to 0.55 μm. It indicates that it is possible to reach lower PSL around Rb = 0.55um. Therefore, sweeping the parameter Rb around 0.55 μm, we find that the discrepancy of absorption between TE and TM modes decreases firstly and then increases with the increase in Rb. At the point Rb = 0.565 μm, a minimum value can be obtained.

**Fig. 5:**
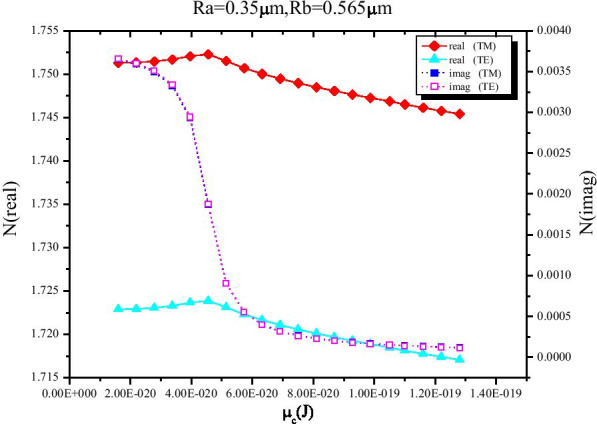
Illustration of the real and imaginary parts of EMI for both TE and TM modes as a function of chemical potential

When Ra = 0.35 μm, Rb = 0.565 μm, wavelength = 2.2 μm, the variation of EMI for both TE and TM modes with chemical potential was analyzed. As shown in Fig. 5, the real part of EMI has same variation trend for TE and TM modes with constant difference. Since the modulator is based on electro-absorption principle, we just need to care about the imaginary part of EMI. What is more, under all the *μ*_c_ values, the *α* of both TE and TM are almost identical. It is the property that we need for designing polarization independent electro-absorption modulator. A highest and lowest value of *α* (proportional to the imaginary part of EMI) can be obtained at *μ*_c_ = 0.1 eV and *μ*_c_ = 0.8 eV, respectively (Fig. 5). Thus, the point of *μ*_c_ = 0.1 eV and *μ*_c_ = 0.8 eV can be chosen as “OFF” and “ON” state point.

**Fig. 6:**
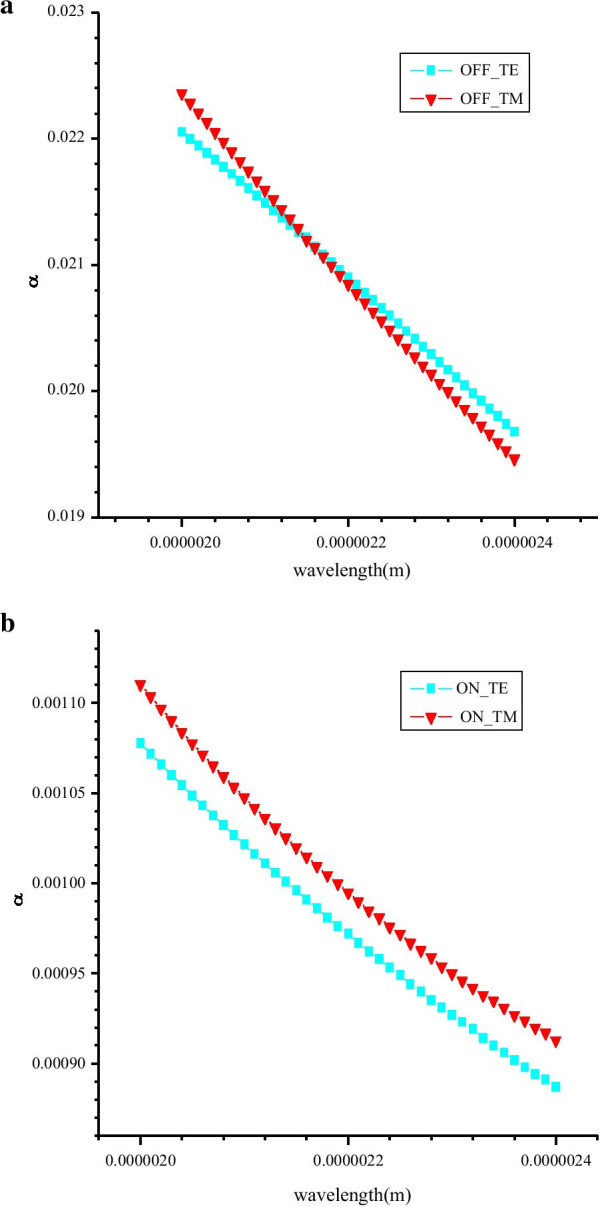
Absorption coefficients (*α*) of TE and TM have an almost identical fluctuation with the change of wavelength at “OFF” state (**a**) and “ON” state (**b**)

**Fig. 7:**
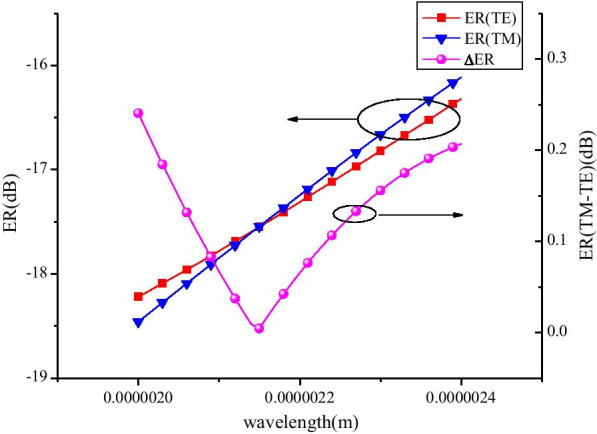
Modulation depth of the two modes and PSL (line ER(TE-TM)) between two modes at different wavelengths

The variation of *α* as a function of wavelength is presented in Fig. 6a, b. It can be seen from Fig. 6 that the *α* of the two modes is very identical with the wavelength change in the strong absorption state (“OFF” state), and the differences between the two modes have been kept relatively small. At the “ON” state, the discrepancy of α between TE and TM modes is at the order of 10^–4^. To measure the discrepancy further and accurately between two modes, PSL is defined as PSL = ER(TE)-ER(TM), where ER is the extinction ratio. We measured the modulation depth of the modulator in two modes as a function of wavelength under the condition of 200 μm long waveguide. As shown in Fig. 7, it can be seen from the diagram that in a wide spectrum range of 2–2.4 μm, the modulation depth of the two modes is more than 16 dB, and PSL is less than 0.24 dB.

For an optical modulator, the 3 dB modulation bandwidth *f*_3dB_ is always one of the important parameters to be concerned about. Since graphene has ultrahigh carrier mobility at room temperature, the graphene-based modulator’s operation speed is no longer limited by minority carrier lifetime like traditional semiconductor modulators are. The *f*_3dB_ of a graphene-based modulator is mainly impeded by RC delay, which can be expressed as

2 $$f_{{3\;{\text{dB}}}} = \frac{1}{2\pi RC}$$

The *R* is the device’s total resistance, including graphene sheet resistance Rs and metal–graphene contact resistance Rc, which has been carefully discussed in previous works [23]-[25]. The *C* is the capacitance of modulator, which mainly consists of the capacitor that is formed by the two graphene flakes. Although this capacitor is not an ideal parallel-plate capacitor model, to preliminarily estimate the *f*_3dB_, we still use the parallel-plate capacitor model to calculate the *C*. In our calculations, Rc = 100 Ω/μm [19] and Rs = 200 Ω/μm [26] were used, and the overlap width of two graphene flakes is about 1.53 μm. The estimated f_3dB_ is as high as 136 GHz. Moreover, lower values of both Rs and Rc are possible in the future, which means higher *f*_3dB_ can be obtained.

**Fig. 8:**
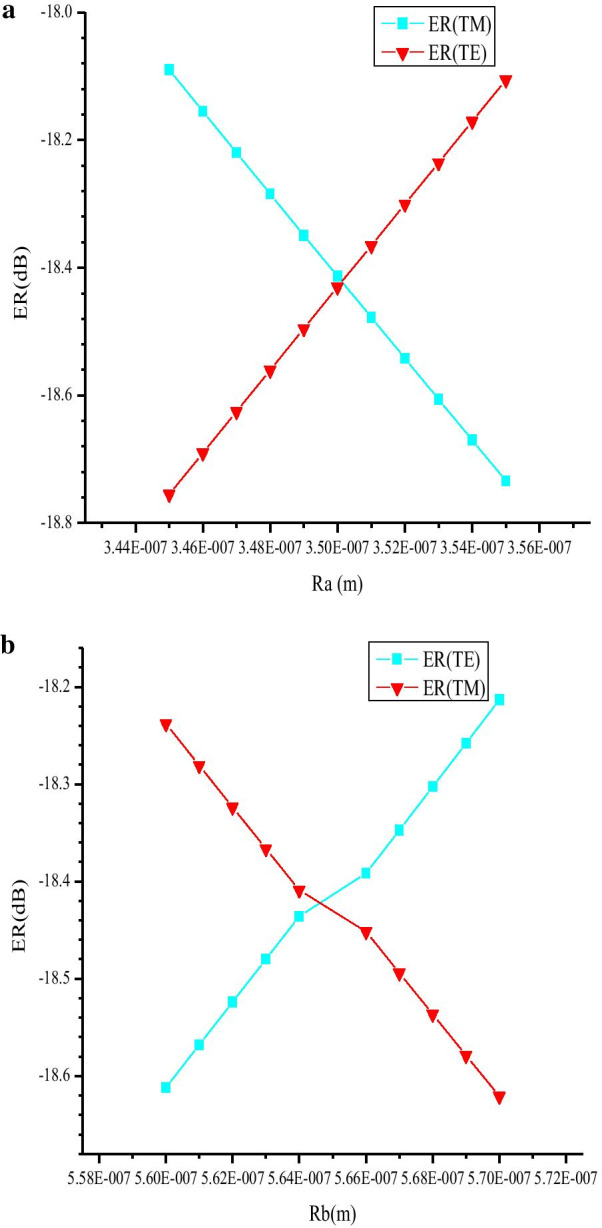
Modulation depth of the two modes at different Ra (**a**) or Rb (**b**)

The above simulations are based on the semiellipse layout with Ra = 0.35 μm and Rb = 0.565 μm. However, in fabrication, this exact radius size cannot always be guaranteed. Therefore, we have also investigated the fabrication tolerance (Fig. 8). When Ra varies from 0.345 to 0.355 μm (Fig. 8a), or Rb varies from 0.56 to 0.57 μm (Fig. 8b), the PSL between two modes is still lower than 0.6 dB. So, our device has large fabrication tolerance.

## Conclusions

In conclusion, we presented a concept of a broadband polarization-independent graphene-based mid-infrared electro-absorption optical modulator. In our structure, a U-structure double-layer graphene is placed in chalcogenide glass waveguide. Under different graphene chemical potentials, different wavelengths and different short radius lengths, the graphene-induced EMI variations for both TE and TM modes are investigated. The results show that TE and TM modes have almost identical absorption coefficient variation in the mid-infrared 2–2.4 μm, which fulfills the requirement of polarization-independent modulation. Based on this structure, the modulator with a length of 200 μm has a modulation depth more than 16 dB. The modulation depth difference between the two modes is 0.24 dB, and the theoretical modulation bandwidth of the device is as high as 136 GHz. We believe that this mid-infrared polarization-independent graphene-based electro-optic modulator will further promote the study of the graphene-based modulator in the middle-infrared bands.

## Data Availability

All data generated or analyzed during this study are included in this published article.

## Abbreviations

ER: Extinction ratio
TM: Transverse magnetic
TE: Transverse electric
PSL: Polarization-sensitivity loss
FEM: Finite element method
EMI: Effective mode index
hBN: Hexagonal boron nitride
